# Supplementary figures and images for: Whole-Exome Sequencing in a Family with an Unexplained Tendency for Venous Thromboembolism: Multicomponent Prediction of Low-Frequency Variant Deleteriousness and of Individual Protein Interaction
Source: Int J Mol Sci. 2023 Sep 7;24(18):13809. doi: 10.3390/ijms241813809 (PMC10530467; doi:10.3390/ijms241813809)

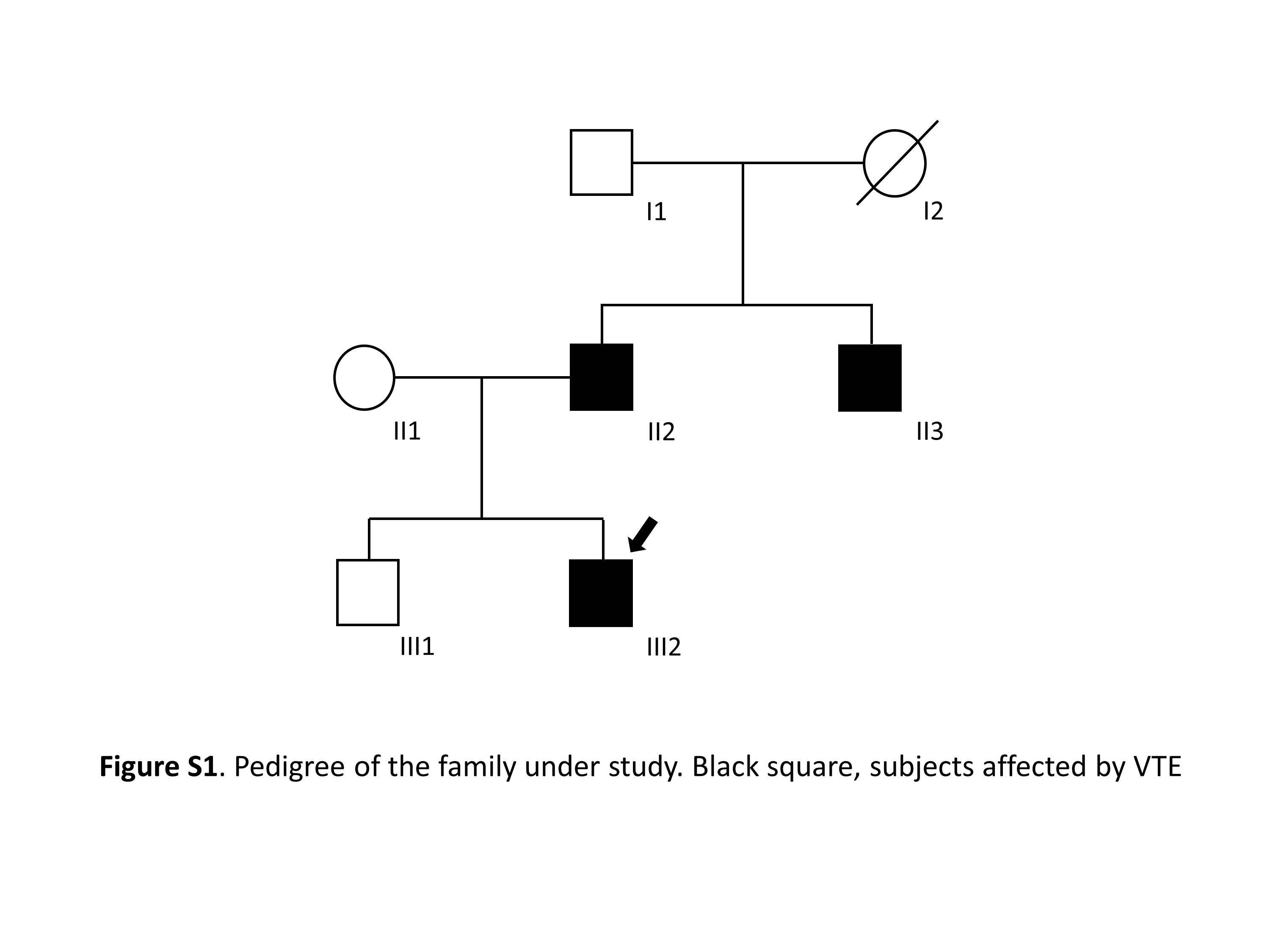

Supplement: Supplementary file 1 [file ijms-24-13809-s001.zip › Figure S1/Figure S1.TIF]
